# Therapeutic antibody activation of the glucocorticoid-induced TNF receptor by a clustering mechanism

**DOI:** 10.1126/sciadv.abm4552

**Published:** 2022-02-25

**Authors:** Changhao He, Rachana R. Maniyar, Yahel Avraham, Roberta Zappasodi, Radda Rusinova, Walter Newman, Heidi Heath, Jedd D. Wolchok, Rony Dahan, Taha Merghoub, Joel R. Meyerson

**Affiliations:** 1 Department of Physiology and Biophysics, Weill Cornell Medical College, New York, NY, USA.; 2 Ludwig Collaborative and Swim Across America Laboratory, Human Oncology and Pathogenesis Program, Memorial Sloan Kettering Cancer Center, New York, NY, USA.; 3 Department of Systems Immunology, Weizmann Institute of Science, Rehovot, Israel.; 4 Parker Institute for Cancer Immunotherapy, Memorial Sloan Kettering Cancer Center, New York, NY, USA.; 5 Weill Cornell Medicine, New York, NY, USA.; 6 Immunology and Microbial Pathogenesis Program, Weill Cornell Graduate School of Medical Sciences, New York, NY, USA.; 7 Leap Therapeutics, Cambridge, MA, USA.; 8 Department of Medicine, Memorial Sloan Kettering Cancer Center, New York, NY, USA.

## Abstract

GITR is a TNF receptor, and its activation promotes immune responses and drives antitumor activity. The receptor is activated by the GITR ligand (GITRL), which is believed to cluster receptors into a high-order array. Immunotherapeutic agonist antibodies also activate the receptor, but their mechanisms are not well characterized. We solved the structure of full-length mouse GITR bound to Fabs from the antibody DTA-1. The receptor is a dimer, and each subunit binds one Fab in an orientation suggesting that the antibody clusters receptors. Binding experiments with purified proteins show that DTA-1 IgG and GITRL both drive extensive clustering of GITR. Functional data reveal that DTA-1 and the anti-human GITR antibody TRX518 activate GITR in their IgG forms but not as Fabs. Thus, the divalent character of the IgG agonists confers an ability to mimic GITRL and cluster and activate GITR. These findings will inform the clinical development of this class of antibodies for immuno-oncology.

## Teaser

Therapeutic antibody agonists for the TNF receptor GITR activate the receptor by mimicking its endogenous ligand.

## INTRODUCTION

The tumor necrosis factor (TNF) receptor superfamily includes 29 receptors that mediate diverse signal transduction pathways involved in immune cell proliferation, apoptosis, and inflammation (*1*–*3*). The glucocorticoid-induced TNF receptor (GITR) is a prominent member of this family because of its central role in regulating T cells to promote immune responses (*4*–*7*). The receptor is a transmembrane protein expressed on the surface of multiple immune cell types including activated effector T cells and is constitutively expressed on regulatory T (T_reg_) cells. It is activated upon engagement with its endogenous ligand (GITRL), which is itself a transmembrane protein displayed on the surface of antigen-presenting cells and endothelial cells (*8*–*10*). Interaction between ligand and receptor occurs at the cell-cell interface and is proposed to generate the formation of high-order GITR arrays that trigger an intracellular signaling cascade (*1*, *11*). The therapeutic rationale and key function of GITR engagement is to inactivate T_reg_ cells that would otherwise promote immune suppression, which has the effect of enhancing immune activity (*6*, *12*). GITR’s central role in immune system regulation has garnered attention for immunotherapeutic cancer treatment and led to the development of anti-GITR antibodies that are proposed to mimic the ability of the endogenous GITRL to cluster receptors into high-order arrays. Anti-GITR antibodies have been successful in multiple tumor models including lymphoma, melanoma, and colon cancer (*13*–*15*) and are undergoing active development and clinical trials in humans (*16*, *17*). However, the precise mechanism of how GITR may be clustered by agonistic antibodies or by its endogenous ligand GITRL is not well understood.

GITR and other TNF receptor family members have been the subject of considerable structural characterization in an effort to understand receptor and ligand structure as well as receptor-ligand interaction and to develop hypotheses about how ligand binding may drive receptor clustering. Sequence conservation in the TNF receptor family is low to moderate, but the general receptor structure features an N-terminal ectodomain that harbors cysteine-rich domains (CRDs) and one transmembrane helix that connects to an unstructured intracellular C-terminal domain (*1*, *3*). Many structures of TNF receptor ectodomains have been reported alone or in complex with ectodomains for their ligands (*1*, *11*, *18*–*20*). However, no structures of full-length TNF receptors have been solved. This knowledge gap is important because it prevents unambiguous determination of the oligomeric state of the receptors, which, in turn, limits our ability to rationalize receptor activation.

Structural work on GITR, GITRL, and the GITR/GITRL complex has focused exclusively on soluble ectodomains from mouse and human orthologs (*11*, *21*–*24*). Structures of mouse GITR (mGITR) and human GITR (hGITR) have shown that, despite low sequence identity (59%), the two ectodomains have nearly identical structures [root mean square deviation (RMSD) < 1 Å] and both ectodomains form monomers in solution (fig. S1) (*11*). Similarly, GITRLs from mouse and human have low sequence identity (52%) and, while individual ligand subunits have highly similar structures (RMSD ~ 2 Å), GITRL forms a dimer in solution (mouse) or a trimer (human) in solution (fig. S1) (*11*, *21*–*24*). As noted, the ectodomains from mGITR and hGITR are monomers in solution (*11*), but in order for the receptor to form higher-order arrays upon binding with its ligand, the receptor must be at least a dimer (fig. S1). Thus, the current molecular picture of GITR does not fully account for its physiological properties. This provides motivation to establish the oligomeric state of GITR and biochemically validate the GITR/GITRL clustering mechanism.

The first GITR-specific agonistic antibody to be developed was the anti-mouse DTA-1 antibody (*6*, *25*), and its in vivo administration can overcome cancer tolerance and induce tumor rejection in several mouse tumor models (*26*–*28*). DTA-1 is proposed to function primarily by targeting mGITR on T_reg_ cells to reduce their immunosuppressive function or by Fc-mediated T_reg_ depletion, which, in turn, promotes antitumor immunity (*15*, *29*). Recently, the anti-hGITR antibody TRX518 was developed as an agonist for hGITR with the ability to reduce the circulating and intratumoral T_reg_ cell populations as well as their suppressive function in an Fc-independent manner (*17*). There are presently no reported structures of an antibody agonist with GITR. In addition, it is unknown whether the divalent nature of immunoglobulin G (IgG) antibodies is essential for agonism or whether monovalent Fab forms can also activate the receptor. These factors limit our ability to interpret the activity of the antibodies, rationally alter existing antibodies, or design altogether new antibodies.

In this study, we investigated mGITR and its interactions with mouse GITRL (mGITRL) and the agonist antibody DTA-1. We hypothesized that mGITR is a dimer and that each subunit can bind a single DTA-1 Fab. By extension, we hypothesized that the IgG form of DTA-1 will cluster together mGITR dimers into an extended receptor antibody array to drive GITR stimulation. We also proposed that the IgG clustering activity is similar to the activity of endogenous mGITRL and that IgG clustering of receptors is essential for antibody agonism. To investigate these hypotheses, we first solved the structure of full-length mGITR in complex with DTA-1 Fab fragments using single-particle cryo–electron microscopy (cryo-EM). We then used purified full-length mGITR and mGITRL along with DTA-1 IgG and Fab to perform analytical binding experiments. Last, we performed cell signaling functional experiments to investigate whether DTA-1 can agonize mGITR in both IgG and Fab forms and whether TRX518 can agonize hGITR in IgG and Fab forms.

## RESULTS

### Structure of mGITR in complex with DTA-1 Fabs

We first sought to understand the structure and organization of full-length mGITR and establish how it interacts with DTA-1 Fab. We hypothesized that mGITR is a dimer because this would be consistent with the proposal that the receptor must be oligomeric to bind multiple mGITRLs or IgG for signaling (fig. S1). We also speculated that each mGITR dimer would interact with two DTA-1 Fabs, rather than interacting with a single Fab (a situation that would result if one Fab sterically occludes binding of a second Fab). To investigate the structure of full-length mGITR, it was necessary to generate protein of sufficient quality and quantity for single-particle cryo-EM. It was also important to fluorescently monitor mGITR during purification and for later biochemical experiments so we fused the receptor to enhanced green fluorescent protein (EGFP) at its C terminus (mGITR_egfp_). The protein was expressed in human embryonic kidney (HEK) 293S cells and then solubilized in *n*-dodecyl-β-d-maltopyranoside (DDM) detergent. The receptor appeared as a sharp monodisperse peak on size exclusion chromatography (fig. S2), indicating that it was suitable for structural study. Purified mGITR_egfp_ was mixed with DTA-1 Fabs to form the mGITR_egfp_/DTA-1 Fab complex.

**Fig. 1.**
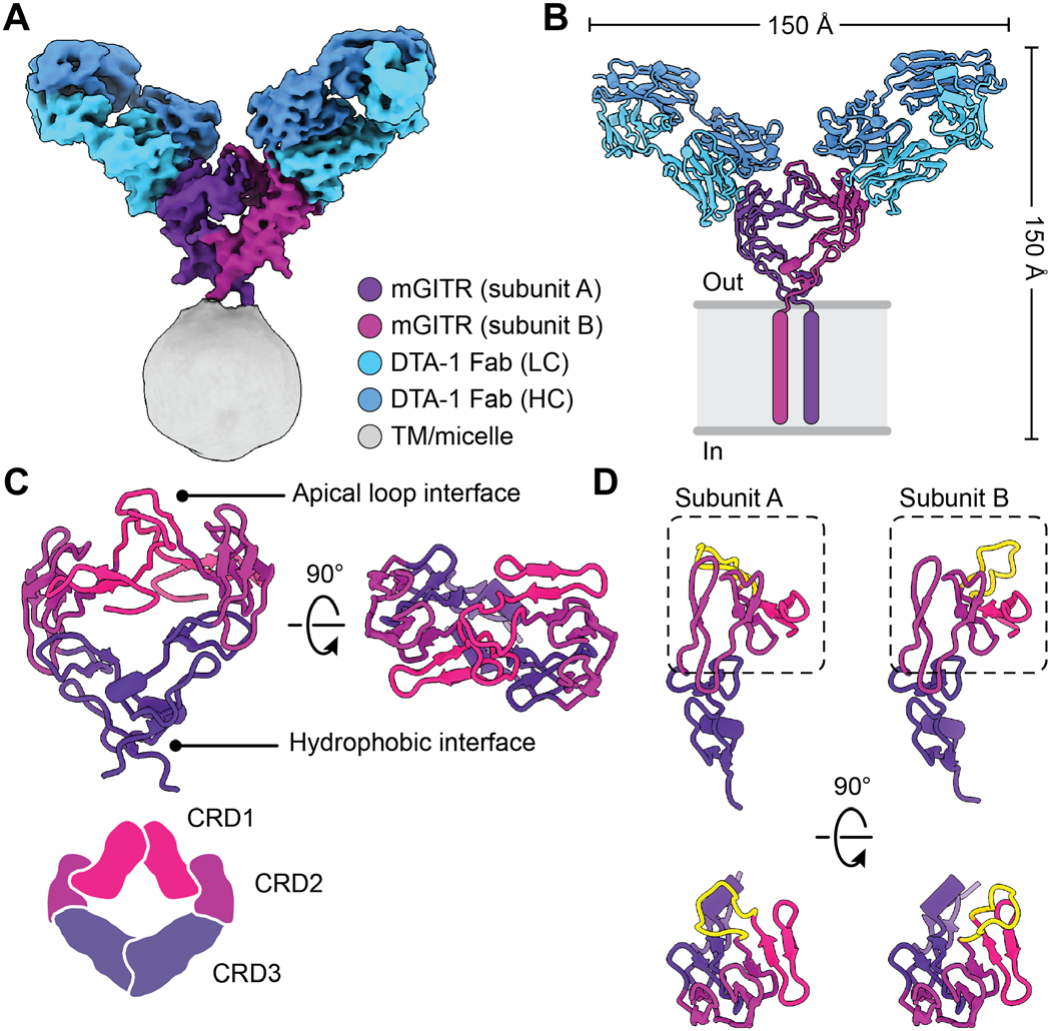
Structure of mGITR in complex with DTA-1 Fabs. (**A**) Cryo-EM density map of mGITR in complex with DTA-1 Fabs. The receptor subunits, Fab heavy chain (HC) and light chain (LC), and micelle are colored as indicated in the legend. (**B**) Molecular model of the mGITR dimer with two Fabs and colored as in (A). The transmembrane helices are illustrated to show the orientation of mGITR with respect to the cell membrane. (**C**) Molecular model of mGITR dimer highlighting the CRD1 (pink), CRD2 (maroon), and CRD3 (purple) regions. The receptor is shown parallel to the membrane (left) and as viewed from the extracellular space (right). (**D**) The two mGITR subunits are shown after separating them and then placing each subunit in the same orientation for comparison. The flexible apical loop of each subunit is highlighted in yellow. The subunits are shown parallel to the membrane (top) and as viewed from the extracellular space (bottom).

The structure of mGITR_egfp_/DTA-1 Fab was solved using single-particle cryo-EM and initially refined to a resolution of ~6 Å. We attributed the modest resolution to the small mass of the complex and the apparent flexibility of the overall receptor-Fab protein complex. However, this limitation was surmounted using further image classification to isolate a more conformationally uniform population of single-particle images, and the mGITR_egfp_/DTA-1 Fab complex was refined to a global resolution of 4.4 Å (Fig. 1A, fig. S3, and table S1). The mGITR subunits and Fab fragments were readily identifiable in the structure, and inspection of the structure revealed that mGITR forms a dimer, with each subunit bound to one Fab. The two transmembrane helices were visible at low resolution in the early stages of refinement and indicated close packing of the helices (fig. S3) but were not well visualized in the final structure. At the measured resolution, the protein backbone and secondary structural features were clearly visualized. Structural analysis was facilitated by docking two copies of the mGITR ectodomain crystal structure [Protein Data Bank (PDB): 7KHX] into the corresponding cryo-EM densities (Fig. 1B). We observed structural asymmetry in the cryo-EM structure at a region we hereto refer to as the “apical loop” of the CRD1 region (Fig. 1C), where the loop conformation on one mGITR subunit did not match the conformation on the second mGITR subunit (Fig. 1D). Notably, this loop was unresolved in the crystal structure of the mGITR ectodomain (*11*), suggesting that the loop may be unstructured when mGITR is in a monomeric form. The C terminus of mGITR and EGFP was not resolved because of the conformational flexibility in the C terminus. The mGITR and DTA-1 Fab regions were modeled as polyalanine chains to match the resolution limit of the cryo-EM map.

### The mGITR dimer is covalently linked by an intersubunit disulfide bond

Analysis of the mGITR dimer shows that each subunit forms a crescent shape, with one dimerization interface proximal to the membrane and the other interface at the apical loops, which are distal to the membrane (Fig. 1C). The dimer interface near the membrane was anticipated from previous crystallographic work and is known to be formed by a group of phenylalanines that create a hydrophobic patch (Fig. 1C) (*11*). However, the interface at the apical loops was unexpected. We wanted to determine what holds the mGITR dimer together and were thus motivated to investigate a possible role for the apical loop interface.

**Fig. 2.**
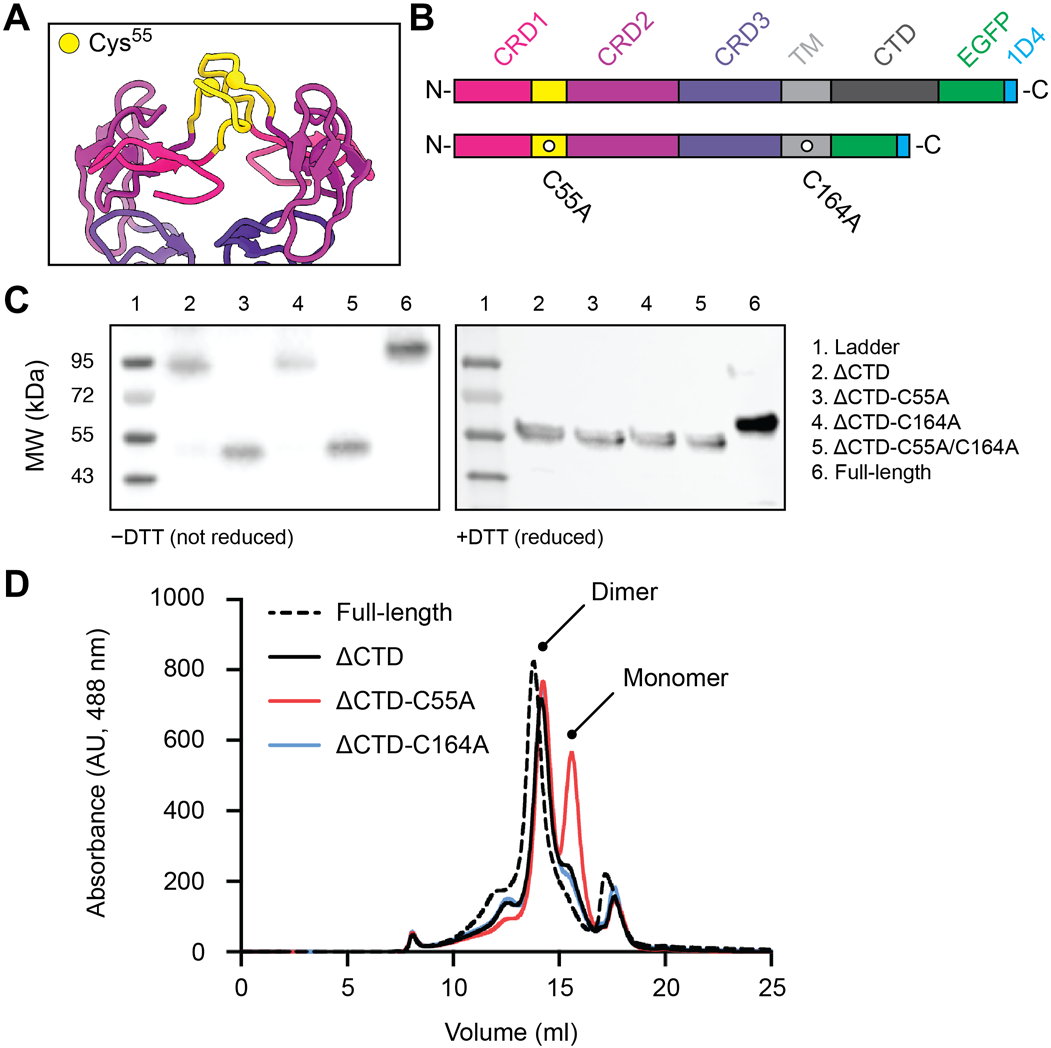
The mGITR dimer interface is locked by an intersubunit disulfide bond. (**A**) mGITR dimer interface with CRD1 (pink), CRD2 (maroon), and CRD3 (purple) and apical loops of CRD1 (yellow). Spheres on the apical loops mark Cys^55^. (**B**) Illustration of the mGITR protein with relevant domains. The full-length protein is shown at the top, and the mGITR_ΔCTD_ construct with C55A and C164A mutations is shown at the bottom. (**C**) Western blots of full-length mGITR and the four CTD deletion constructs. Nonreduced (−DTT) and reduced (+DTT) conditions are on the left and right, respectively. Each lane is numbered according to the sample it contains, and sample descriptions are provided in the legend. MW, molecular weight. (**D**) FSEC traces showing EGFP fluorescence of lysate from HEK293 cells expressing full-length mGITR_egfp_ (dashed line), mGITR_ΔCTD-egfp_ (solid black line), mGITR_ΔCTD-egfp_-C55A (red line), and mGITR_ΔCTD-egfp_-C164A (blue line). AU, arbitrary units.

During structural analysis, we noted that the apical loop on each mGITR subunit harbors an apparently unpaired cysteine at position 55. This is particularly conspicuous because TNF receptors, including GITR, are replete with cysteines, which are typically disulfide-bonded. We observed that Cys^55^ residues on both apical loops are in close proximity (Fig. 2A) and visualized density between the subunits, which was suggestive of a disulfide bond (fig. S3G). Accordingly, we hypothesized that Cys^55^ forms an intersubunit disulfide bond and designed a series of mutants and analyzed them using Western blots. To narrow the focus of our experiments, we removed the unstructured C-terminal residues (C186-P228) from mGITR because the region contains three cysteines that are uncharacterized. The resulting construct has a total of 17 cysteines, retains EGFP at the C-terminal end, and is referred to as mGITR_ΔCTD-egfp_ (Fig. 2B). Of the remaining 17 cysteines in the construct, 14 are established to form the seven intrasubunit disulfide bonds within each mGITR ectodomain (*11*), which left 3 cysteines to consider. Cys^14^ is part of the predicted signal peptide and so is not present in the mature protein. The remaining two cysteines are Cys^55^ in the apical loop and Cys^164^ in the transmembrane helix. To interrogate these two residues, we made the single mutants mGITR_ΔCTD-egfp_-C55A and mGITR_ΔCTD-egfp_-C164A and the double mutant mGITR_ΔCTD-egfp_-C55A/C164A (Fig. 2B). All constructs encode a C-terminal 1D4 tag (*30*) following EGFP, which allowed for analysis of lysate by anti-1D4 Western blot.

Full-length mGITR_egfp_ and mGITR_ΔCTD-egfp_ appeared as dimers and could be shifted to monomer positions upon reducing treatment with dithiothreitol (DTT) (Fig. 2C). This confirmed the presence of intersubunit disulfide bonds in the receptor and showed that they must form at C55, C164, or both positions. This was further confirmed by analysis of the C55A/C164A double mutant, which migrated as a monomer without addition of DTT (Fig. 2C). We next analyzed the C55A mutant and found that it migrated to the monomer position with or without DTT addition, supporting the hypothesis that Cys^55^ forms a disulfide bond between the two mGITR subunits (Fig. 2C). This is consistent with recent work reporting the role of Cys^55^ in dimer stability (*31*). Last, the C164A mutant migrated as a dimer without DTT, showing that no disulfide bond forms in the transmembrane region (Fig. 2C).

In addition, we exploited the EGFP tag on the constructs to perform fluorescence size exclusion chromatography (FSEC) experiments (*32*). This allowed us to further examine the effect of mutations on dimerization but without subjecting the protein to the denaturing conditions of SDS–polyacrylamide gel electrophoresis (SDS-PAGE) that disrupts noncovalent interactions. The proteins were expressed in HEK293 cells and solubilized with DDM, and the lysate was injected into a size exclusion column equipped with a fluorescence detector. The EGFP signal from the lysate showed a sharp dimer elution peak for full-length mGITR_egfp_, and the peak for mGITR_ΔCTD-egfp_ was shifted slightly to the right, reflecting its smaller mass (Fig. 2D). Comparison of mGITR_ΔCTD-egfp_ with the C55A and C164A mutants showed the appearance of a second peak for C55A but not for C164A (Fig. 2D). On the basis of the elution position, the second peak is smaller in mass than the first peak and is likely an mGITR monomer. This experiment supports the idea that Cys^55^ contributes to dimer stability, but the fact that the C55A condition also retains a clear dimer peak suggests that Cys^55^ is not the only contributor to dimer stability. The hydrophobic patch near the transmembrane region is likely the other major contributor (Fig. 1C) (*11*).

### Determinants of DTA-1 discrimination between mGITR and hGITR

**Fig. 3.**
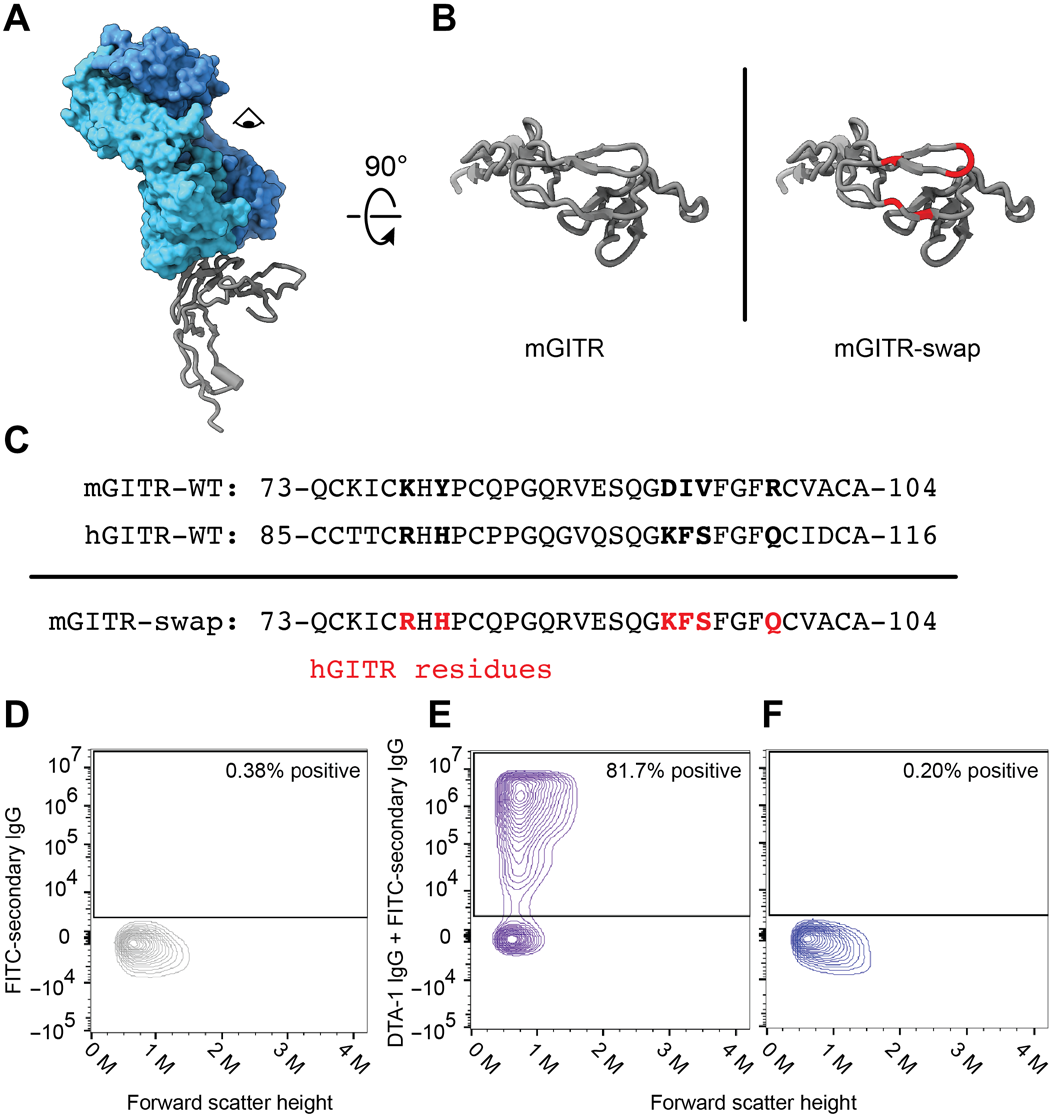
Human and mouse agonistic antibodies bind distinct residues on one epitope. (**A**) A single mGITR subunit (gray) bound to a DTA-1 Fab with light chain (light blue) and heavy chain (dark blue). (**B**) Extracellular view of an mGITR subunit (left) and mGITR with residues proximal to the DTA-1 Fab and swapped to hGITR residues (right). (**C**) Sequence alignment between mGITR and hGITR in the region where DTA-1 binds mGITR. The residues on mGITR that are swapped to hGITR residues are indicated in the mGITR-swap sequence. WT, wild type. (**D** to **F**) Flow cytometry of cells expressing wild-type mGITR and treated with no primary antibody (negative control) (D), cells expressing wild-type mGITR and treated with DTA-1 IgG (E), and cells expressing the mGITR-swap mutant and treated with DTA-1 IgG (F). A fluorescein isothiocyanate (FITC)–labeled secondary antibody was used in all three experiments. The percentages correspond to the fraction of cells reporting an FITC fluorescent signal.

The mGITR/DTA-1 structure showed that DTA-1 binds an exposed patch on the mGITR ectodomain that is entirely on CRD2 (Fig. 3, A and B). The patch of residues includes Lys^78^, Tyr^80^, Asp^93^, Ile^94^, Val^95^, and Arg^99^. These six residues are particularly conspicuous because they are not conserved in hGITR. This led us to hypothesize that these residues are essential for DTA-1 binding and that mutating the residues to their human equivalents would abolish binding of DTA-1 to mGITR. To investigate this, we used flow cytometry, which allowed us to interrogate the receptor-antibody interaction on the cell surface. As a control, we verified that DTA-1 binds cells expressing wild-type mGITR (Fig. 3E). We then swapped the six target residues on mGITR for their human equivalents to generate the mGITR-swap mutant (Fig. 3C) and performed flow cytometry (Fig. 3F). Results on mGITR-swap showed that the mutant loses the ability to bind DTA-1. These data experimentally validate the mGITR/DTA-1 interaction observed in the structure and reveal residues involved in conferring DTA-1 specificity for mGITR.

### mGITRL and DTA-1 drive clustering of mGITR

**Fig. 4.**
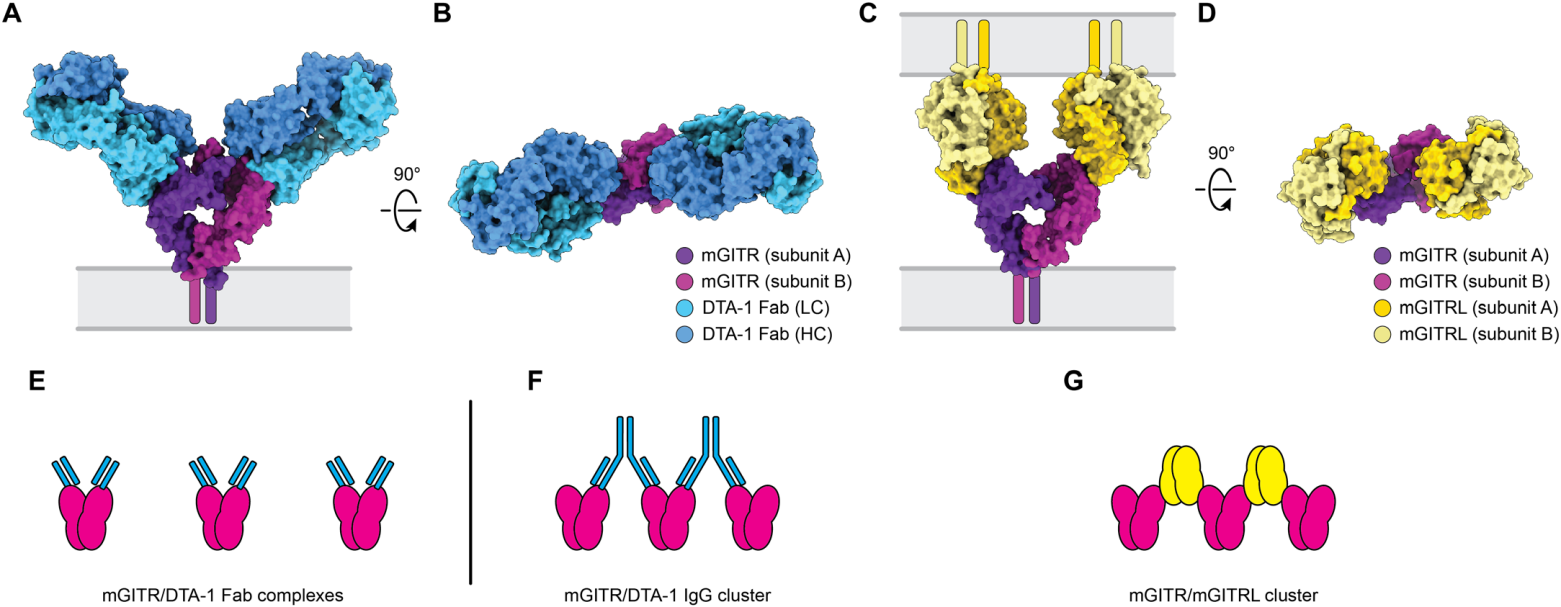
DTA-1 and mGITRL bind overlapping epitopes on mGITR. (**A** and **B**) Surface rendering of the mGITR/DTA-1 Fab molecular model colored as indicated in the legend. (**C** and **D**) Proposed molecular arrangement of the mGITR dimer in complex with two copies of the mGITRL dimer. This model was built using the mGITR dimer structure (present study) as a template and aligning two copies of the mGITR monomer/mGITRL monomer cocrystal structure to the dimer. The effect was to create a model of the mGITR dimer with one mGITRL monomer bound to each mGITR subunit. Last, two copies of the mGITRL dimer were aligned to each mGITRL monomer to generate the final model presented in (C) and (D). The model is colored as indicated in the legend. (**E**) Illustration of discrete complexes formed by mGITR dimers with DTA-1 Fabs. (**F**) Illustration of clusters formed by mGITR dimers with DTA-1 IgG. (**G**) Illustration of clusters formed by mGITR dimers with mGITRL.

We next sought to understand if DTA-1 IgG can cluster multiple receptor dimers and the extent to which it competes with mGITRL for binding to the receptor. To initiate the analysis, we generated a model of the mGITR dimer bound to mGITRL. This was made possible by using the co-complex crystal structure of the mGITR ectodomain monomer bound to the mGITRL ectodomain dimer (*11*). With the mGITR dimer structure from cryo-EM as a reference (present study), we used structural alignment to position two copies of the receptor/ligand structure together. The result is a model of the mGITR dimer with each mGITR subunit bound to mGITRL (Fig. 4). Comparison of this mGITR/mGITRL model to our mGITR/DTA-1 Fab structure showed that the DTA-1 Fab binding area overlaps with the ligand binding area. In addition, the binding orientation of ligand and Fab suggested that a single mGITRL dimer (divalent) or a single DTA-1 IgG (divalent) should be capable of “bridging” two mGITR dimers (Fig. 4). This is consistent with the hypothesis that agonistic antibodies for TNF receptors mimic the activity of native ligands by clustering receptors (*1*, *33*).

To investigate DTA-1 IgG and mGITRL clustering, we performed a series of binding experiments using purified full-length mGITR_egfp_, full-length mGITRL, DTA-1 IgG, and DTA-1 Fab. The experimental platform we used for binding experiments was FSEC (*32*). The rationale is that, because mGITR_egfp_ emits an EGFP signal, we could readily monitor its change in elution position when mixing the receptor with different binding partners that lack EGFP tags. As controls, all experiments were also performed while monitoring tryptophan fluorescence, which is advantageous for simultaneously monitoring the elution positions of all protein species in the experiment but is ambiguous when multiple elution peaks overlap (fig. S4). Because mGITR_egfp_ and mGITRL are full-length proteins with transmembrane regions, the experiments were done with DDM detergent in the buffer to maintain the protein integrity and solubility.

**Fig. 5.**
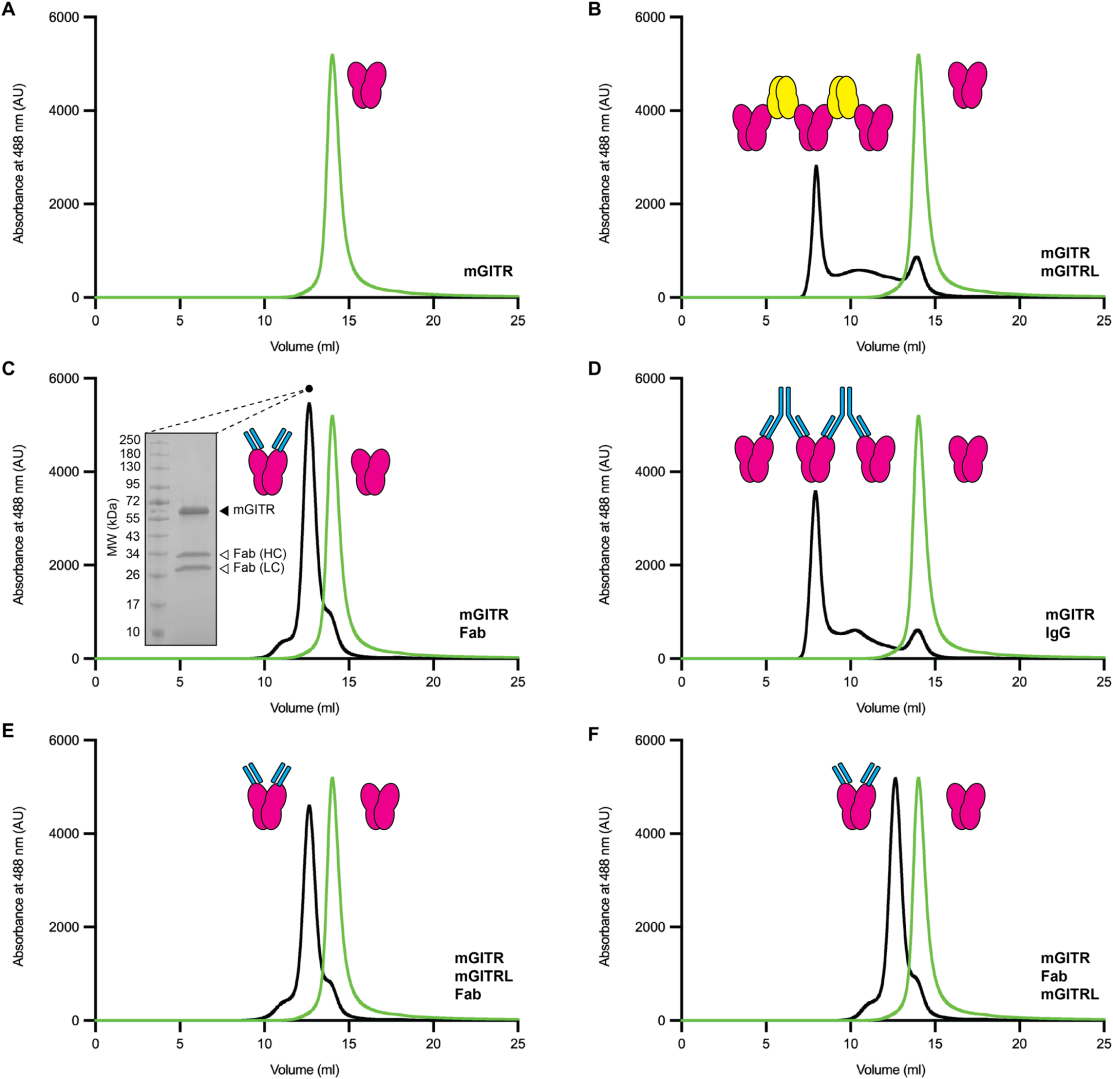
DTA-1 mimics the receptor clustering of mGITRL. (**A** to **F**) FSEC traces of mGITR fused to EGFP (mGITR_egfp_). The molar ratio used throughout is 1:15:4:2 for mGITR_egfp_:mGITRL:Fab:IgG. Each trace shows mGITR_egfp_ alone (green) and the resulting trace from mGITR_egfp_ mixed with another protein or proteins (black). The traces are mGITR_egfp_ alone (A), mGITR_egfp_ with mGITRL (B), mGITR_egfp_ with DTA-1 Fab (C), mGITR_egfp_ with DTA-1 IgG (D), mGITR_egfp_ incubated (5 min, 4°C) with mGITRL before adding DTA-1 Fab (E), and mGITR_egfp_ incubated with DTA-1 Fab before adding mGITRL (F). The inset in (C) shows isolation of the mGITR_egfp_-Fab peak and analysis by SDS-PAGE with bands for mGITR_egfp_, Fab heavy chain, and light chain. Cartoon illustrations show mGITR (magenta), mGITRL (yellow), DTA-1 IgG (cyan) (large symbol), and DTA-1 Fab (cyan) (small symbol).

We first tested injection of mGITR_egfp_ alone, which showed a sharp monodisperse peak at ~14 ml (Fig. 5A). When mGITR_egfp_ was mixed with mGITRL, the primary elution peak shifted to ~8 ml (Fig. 5B). This leftward shift indicated a large increase in mass, which we interpret as the formation of the mGITR_egfp_-mGITRL complex. We also note that the ~8-ml elution position is in the “void” range of the column, which is the range beyond the resolution limit of the column. In essence, this peak position suggests a large complex of undefined mass. We interpreted this to be an extended array of mGITR_egfp_-mGITRL and it offered support for the hypothesis that mGITRL clusters mGITR_egfp_.

We next examined the ability of DTA-1 to mimic the mGITRL-induced clustering of mGITR_egfp_. As a control, we first tested a mixture of DTA-1 Fab with mGITR_egfp_ with the expectation that it would give a leftward shift in elution position but would remain within the resolution limit of the column. The signal shifted from ~14 ml for mGITR_egfp_ alone to ~12 ml for mGITR_egfp_ with DTA-1 Fab (Fig. 5C). This signaled the formation of a complex larger than mGITR_egfp_ alone but smaller than the mGITR_egfp_-mGITRL complex (Fig. 5B). To further validate the Fab-induced shift, we sampled the column fraction corresponding to the shifted peak (12 ml) and analyzed it on SDS-PAGE. This showed clear bands for mGITR_egfp_ and for the DTA-1 Fab heavy and light chains (Fig. 5C) and verified our interpretation of the shift in elution position. We tested IgG-induced clustering by mixing mGITR_egfp_ with DTA-1 IgG and recorded the FSEC trace (Fig. 5D). This experiment yielded an elution profile that closely matched the mGITR_egfp_-mGITRL profile (Fig. 5B), suggesting that both mGITRL and DTA-1 IgG induce the formation of large extended arrays of mGITR_egfp_.

Last, we sought to understand whether the mGITRL-induced clustering of mGITR_egfp_ is reversible or whether the resulting assembly is merely a soluble aggregate. To test this, we incubated mGITR_egfp_ and mGITRL to form the protein assembly and then added DTA-1 Fab and injected the sample onto the size exclusion column. The resulting elution profile (Fig. 5E) matched the profile of mGITR_egfp_ with DTA-1 Fab (Fig. 5C). As an additional test, we instead added Fab first and then mGITRL (Fig. 5F), and the result again matched the profile from mGITR_egfp_ with DTA-1 Fab (Fig. 5C). These results give a clear indication that DTA-1 Fab can reverse mGITR_egfp_-mGITRL clustering. Furthermore, given the vast excess of mGITRL used in the experiment (fig. S4), it shows that DTA-1 binds with higher affinity than mGITRL.

### Agonistic DTA-1 and TRX518 function in IgG form but not Fab form

Our structural data show that mGITR is a dimer and that each subunit can bind a DTA-1 Fab (Fig. 1). Biochemical binding data validate this structural model and demonstrate that DTA-1 IgG can cluster purified mGITR dimers in an extended array, while DTA-1 Fab cannot (Fig. 5). On the basis of these observations, we hypothesized that DTA-1 clustering of mGITR may be essential to its agonistic activity. Furthermore, we also hypothesized that the same mechanism underpins the activity of the anti-hGITR antibody TRX518 (*17*).

**Fig. 6.**
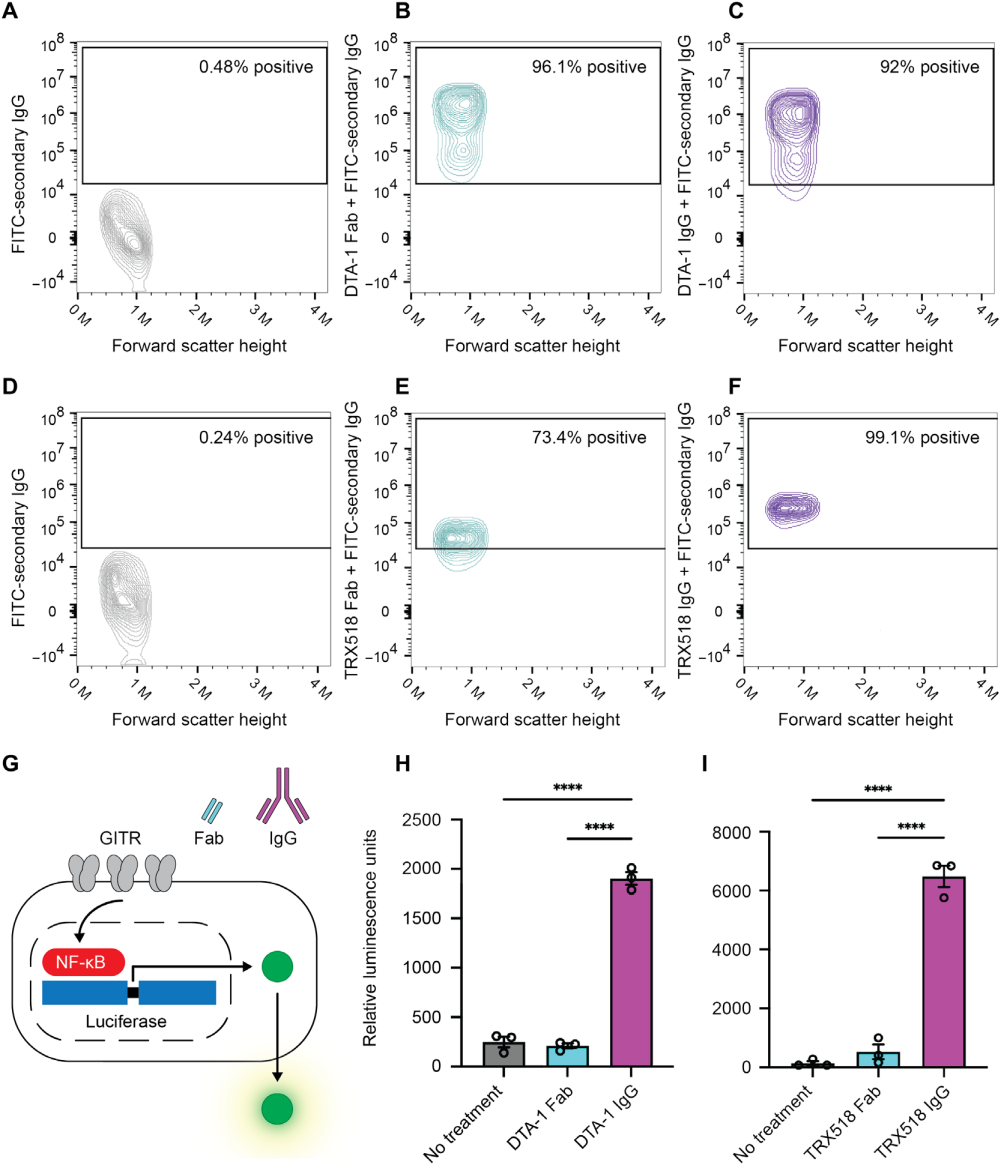
Full-length IgG is required for GITR agonism. (**A** to **C**) Flow cytometry experiments with mGITR and no primary antibody (negative control) (A), mGITR with DTA-1 Fab (B), and mGITR with DTA-1 IgG (C). (**D** to **F**) Flow cytometry experiments with hGITR and no primary antibody (negative control) (D), hGITR with TRX518 Fab (E), and hGITR with TRX518 IgG (F). (**G**) Illustration of luciferase assay used to measure GITR activation by DTA-1 and TRX518 Fab and IgG. (**H**) Luciferase assay measuring mGITR activation in cells that are untreated or treated with DTA-1 Fab or DTA-1 IgG. (**I**) Luciferase assay measuring hGITR activation in cells that are untreated or treated with TRX518 Fab or TRX518 IgG. One-way analysis of variance (ANOVA) followed by a Tukey’s multiple comparison test was used to determine statistical significance between groups (****P < 0.0001).

We first performed a flow cytometry experiment to confirm that DTA-1 Fab and IgG bind mGITR to similar extents (Fig. 6, A to C). Likewise, we tested TRX518 Fab and IgG to verify their binding to hGITR (Fig. 6, D to F). To compare the agonistic ability of the Fabs and IgGs, we expressed either mGITR or hGITR in an HEK293 cell line containing a nuclear factor κB (NF-κB) luciferase reporter system. GITR and other TNF receptors signal via NF-κB (*34*), and in this assay, that signaling drives luciferase expression, which is measured as a luminescence readout (Fig. 6G). Because an IgG is divalent and a Fab is monovalent, the experiments compared Fab and IgG at a 2:1 molar ratio to ensure that the samples have identical binding potential. Addition of DTA-1 Fab to mGITR-expressing cells showed no significant difference in luminescence above untreated cells (Fig. 6H). In contrast, DTA-1 IgG showed a significant increase in luminescence, approximately six times the level observed for the Fab, which demonstrated successful downstream NF-κB activation (Fig. 6H). Experiments with TRX518 Fab and IgG yielded an analogous result, with Fab exhibiting little capacity to stimulate hGITR and TRX518 IgG driving significant stimulation (Fig. 6I). These results provide clear evidence that an IgG framework is required for DTA-1 and TRX518 agonism of GITR.

## DISCUSSION

In this study, we solved the structure of a full-length TNF receptor, mGITR, in complex with the agonistic antibody DTA-1 Fab (Fig. 1). The structure illuminates the oligomeric state of the receptor, showing that it forms a dimer stabilized by a hydrophobic patch near the membrane interface (*11*) and by an unexpected intersubunit disulfide bond at the apex of the receptor ectodomain (Fig. 2). Each subunit of the dimer interacts with a DTA-1 Fab (Fig. 1), and the binding interface was verified by point mutations and flow cytometry (Fig. 3). We performed binding experiments with purified full-length mGITR_egfp_ and mGITRL, as well as DTA-1 IgG and Fab. These experiments showed the ability of mGITRL and DTA-1 IgG to cluster mGITR into extended arrays and that DTA-1 competes with and displaces mGITRL from its binding site on mGITR (Fig. 5). We used cell-based functional assays to compare DTA-1 and TRX518 antibodies and show that they function in their IgG forms but not as Fabs (Fig. 6).

A key question is whether hGITR is also a dimer. The fact that mGITR and hGITR ectodomains have virtually identical crystallographic structures (fig. S1) hints that the full-length structures might also be similar. The cysteine involved in the intersubunit disulfide bond we found at the mGITR dimer interface (Cys^55^) (Fig. 2) is conserved in hGITR (Cys^57^), which argues that hGITR also oligomerizes at this position and forms a dimer. An important question is whether the mGITR/mGITRL system can be used as a model for clinical testing and development of human therapeutics, and this warrants further investigation and should be taken into consideration when using preclinical models. The oligomeric state of TNF receptors has long been debated, with hypotheses put forward for monomeric, dimeric, and trimeric configurations depending on the functional state of the receptor (*1*, *35*, *36*). Knowledge of the actual oligomeric state or states of TNF receptor family members has profound implications for how the receptors signal and thus how to therapeutically modulate their signaling. The mGITR dimer structure reported here provides a template to consider the wider TNF receptor family. However, given the sequence and structural variation in the family, there is likely considerable organizational diversity among full-length TNF receptor structures.

Together, our structural work, binding experiments, and cell-based functional experiments support the conclusion that DTA-1 and TRX518 must be in an IgG form to drive optimal signaling of GITR (Figs. 1, 5, and 6). This strongly suggests that the divalent nature of IgG antibodies is integral to their agonism and that receptor clustering is needed to stimulate the GITR signaling pathway. This insight will be important to integrate into efforts to fine-tune GITR signaling, and it will be essential to establish whether agonistic antibodies targeting other TNF receptors such as OX40 or 4-1BB agonize those systems with a similar mechanism. It will also be valuable to determine the oligomeric state of these receptors and define their interaction with agonistic antibodies. For example, this approach could improve the mechanistic view on the differences between the anti–4-1BB agonistic antibodies urelumab and utomilumab (Pfizer) (*33*). Our study also highlights the question of how TNF receptor antibody antagonists may function. To this end, it will be valuable to investigate a TNF receptor system for which both agonist and antagonist antibodies have been developed, such as TNFR2 (*37*, *38*).

## MATERIALS AND METHODS

### Expression and purification of DTA-1 and generation of DTA-1 Fabs

Anti-mGITR clone DTA-1 was generated as a human IgG1 subclass. The sequences of the variable regions of the heavy and light chains (provided by H. Alvarez-Jares, AbbVie Biotherapeutics Inc.) were synthesized by BioBasic, polymerase chain reaction (PCR)–amplified, and cloned into mammalian expression vectors in frame with the constant regions of the human IgG1 heavy chain or human kappa light chain, respectively. Plasmid sequences were validated by direct sequencing (Life Sciences Core Facility, Weizmann Institute of Science). To produce antibodies, antibody heavy and light chain expression vectors were transiently transfected into Expi293 cells (Thermo Fisher Scientific). The secreted antibodies in the cell supernatant were purified using Protein G Sepharose 4 Fast Flow Resin (GE Healthcare). Purified antibodies were dialyzed in phosphate-buffered saline and sterile-filtered (0.22 μm). Purity was assessed by SDS-PAGE and Coomassie staining and was estimated to be >90%. DTA-1 Fab fragments were generated by papain cleavage and purified using the Pierce Fab Preparation Kit (Thermo Fisher Scientific).

### Expression and purification of mGITR

The gene for mGITR was cloned into the pEZT-BM expression vector (*39*). A thrombin cleavage site, EGFP, and an 8× histidine tag were added in frame at the C terminus. Receptor protein was produced using the BacMam method (*40*). Following this method, the mGITR expression construct was transformed into DH10Bac cells to generate bacmid, which was used to produce P1 baculovirus by transfecting Sf9 insect cells in ESF 921 medium (Expression Systems) and further amplified to P2 baculovirus. Flasks of HEK293S GnTI^−^ suspension cells (American Type Culture Collection, catalog number CRL-3022) were cultured in FreeStyle suspension medium (Gibco) supplemented with 2% fetal bovine serum (Gibco) and Anti-Anti (Gibco) at 37°C and 8% CO_2_, and 5% (v/v) P2 virus was added when the suspension cells reached a density of 3.5 × 10^6^ cells/ml. At 16 hours after transduction, sodium butyrate (Sigma-Aldrich) was added into the suspension cells to a final concentration of 10 mM and the cells were shifted to 30°C and 8% CO_2_. At 72 hours after transduction, cells were pelleted down by centrifugation at 6200*g* for 20 min, and at 4°C, and pellets were flash-frozen in liquid nitrogen and stored at −80°C.

The whole purification was performed on ice or in the cold room at 4°C. Thawed cell pellets were resuspended in buffer containing 20 mM tris (pH 8.0), 300 mM NaCl, 0.8 μM aprotinin, leupeptin (2 μg/ml), 2 μM pepstatin, 0.5 mM EDTA, 1 mM phenylmethylsulfonyl fluoride (PMSF), and deoxyribonuclease (25 μg/ml). The cells were sonicated until homogeneous, and the lysates were clarified by centrifugation at 7200*g* for 20 min and further clarified by ultracentrifugation at 125,000*g* for 2 hours. The membrane pellet was resuspended by Dounce homogenization in buffer containing 20 mM tris (pH 8.0), 300 mM NaCl, 0.8 μM aprotinin, leupeptin (2 μg/ml), 2 μM pepstatin, 0.5 mM EDTA, and 1 mM PMSF. An equal volume of the same buffer containing 100 mM DDM (Anatrace) was added into the mixture for a final DDM concentration of 50 mM. The sample was mutated for 1 hour and then ultracentrifuged at 125,000*g* for 50 min. The supernatant was filtered through a 0.45-μm filter and loaded on pre-equilibrated TALON resin (Takara) with purification buffer containing 20 mM tris (pH 8.0), 150 mM NaCl, and 0.5 mM DDM. The resin was washed with three column volumes of purification buffer containing 10 mM imidazole and eluted with three column volumes of purification buffer containing 40 and 250 mM imidazole, respectively. The main elution fractions were concentrated to 700 μl using a centrifugal filter unit [10-kDa molecular weight cutoff (MWCO), Amicon] and injected on a Superose 6 Increase 10/300 GL column (Cytiva) equilibrated with purification buffer. The peak fractions were verified by SDS-PAGE gel and then pooled and concentrated. To isolate the mGITR/DTA-1 Fab complex, mGITR was mixed with Fab in a ratio of 1:3 (w/w) and injected on a Superose 6 Increase 10/300 GL column equilibrated with purification buffer. The peak fractions for mGITR/DTA-1 Fab complex were collected, analyzed by SDS-PAGE, and concentrated using a centrifugal filter (100-kDa MWCO).

### Cryo-EM sample preparation and data acquisition

Cryo-EM samples were prepared by adding a 3-μl droplet of mGITR/DTA-1 Fab complex (4 mg/ml) to plasma-treated UltrAuFoil 1.2/1.3 300 mesh grids (Quantifoil), followed by blotting and plunge-freezing using Vitrobot Mk IV (Thermo Fisher Scientific) set for 2-s blot time and −5 blot force. Samples were imaged on Talos Arctica (Thermo Fisher Scientific) operated at 200 kV and ×36,000 nominal magnification and equipped with a Gatan K3 camera. Movies were acquired in superresolution mode (0.548 Å pixel size) with a nominal defocus range of 1.1 to 2.9 μm. Each movie was 40 frames and had a total exposure time of 2.8 s and an accumulated dose of 53.10 to 57.04 e^−^/Å^2^. A total of 12,818 movies were collected. Data collection was managed using Leginon (*41*).

### Image processing

Movie stacks were corrected for beam-induced motion and dose-weighted in RELION 3.1 (*42*) with twofold binning, yielding an image pixel size of 1.096 Å. These images were used for contrast transfer function (CTF) estimation with CTFFIND4.1 (*43*). From a data subset, about 3500 particles were manually picked and extracted with a box size of 384 pixels to generate a three-dimensional (3D) initial model for 3D reference-based autopicking in RELION 3.1. A total of 3,152,686 particles were autopicked, extracted with a box size of 384, and then imported into cryoSPARC v3.1.0 (*44*). One round of ab initio reconstruction with four classes and no symmetry imposed followed by several rounds of heterogeneous refinement were performed to obtain a homogeneous set of particles. After another round of ab initio reconstruction with two classes and no symmetry imposed, and one round of heterogeneous refinement followed by nonuniform refinement, a final dataset of 423,425 particles was reconstructed to produce a 4.4-Å resolution map.

### Model building and validation

The model for mGITR/DTA-1 was built starting with the crystal structure of the extracellular domain of mGITR (PDB: 7KHX). Two copies of this model were docked into the cryo-EM density map using UCSF Chimera (*45*) and then imported into COOT (*46*). The loop region between Tyr^48^-Lys^57^ was missing from the crystal structure and was modeled using the cryo-EM map. This region includes a disulfide bond at C55, which linked the two mGITR subunits. To model the DTA-1 Fabs, a homology model was generated with SWISS-MODEL (*47*) from the crystal structure of a mouse Fab (PDB: 6DWA). The overall correspondence between the Fab homology model and the map was strong, except for the variable loops in the ranges Ile^37^-Val^67^, Gly^84^-Leu^95^, and Gln^20^-Ser^30^ near the Fab interface with mGITR, which were accordingly remodeled to match the density map. The map did not provide sufficient constraints to model all side chains, so all residues except disulfide-bonded cysteines were stubbed to alanine in the final step of modeling.

### Expression and purification of mGITRL

The gene for mGITRL was cloned into the pEZT-BM expression vector (*39*) with a Strep tag added in frame at the N terminus. GITRL protein was produced in the same way as for the receptor protein except 10% (v/v) P2 virus was added when the suspension cells reached a density of 3.5 × 10^6^ cells/ml and cells were pelleted down at 96 hours after transduction. The whole purification was performed on ice or in the cold room at 4°C. The membrane pellet and the lysate supernatant were prepared the same way as for the receptor. The lysate supernatant was filtered through a 0.45-μm filter and loaded on a pre-equilibrated StrepTrap HP column (Cytiva) with purification buffer containing 20 mM tris (pH 8.0), 300 mM NaCl, and 0.5 mM DDM. The column was washed with 10 column volumes of purification buffer and eluted with 5 column volumes of purification buffer containing 20 mM desthiobiotin (IBA). The main elution fractions were concentrated to 700 μl using a centrifugal filter unit (10-kDa MWCO, Amicon) and injected on a Superose 6 Increase 10/300 GL column (Cytiva) equilibrated with purification buffer. The peak fractions were verified by SDS-PAGE gel and then pooled and concentrated.

### FSEC experiments

FSEC experiments were performed with either cell lysate or purified proteins. In all experiments, a high-performance liquid chromatography (HPLC) with autosampler (Shimadzu) was connected to a Superose 6 Increase 10/300 GL column (Cytiva) followed by a fluorescence detector (Shimadzu).

For lysate experiments, HEK293 cells expressing mGITR constructs fused to EGFP were resuspended in buffer containing 20 mM tris (pH 8.0), 300 mM NaCl, and 50 mM DDM and extracted at 4°C with gentle rocking for 1 hour. Lysate was clarified by ultracentrifugation before injecting into the HPLC running buffer containing 20 mM tris (pH 8.0), 300 mM NaCl, and 0.5 mM DDM. EGFP fluorescence was monitored using excitation and emission wavelengths of 488 and 509 nm, respectively.

For experiments with purified proteins, samples were run on the HPLC buffer containing 20 mM tris (pH 8.0), 300 mM NaCl, and 0.5 mM DDM. To monitor EGFP fluorescence, excitation and emission wavelengths of 488 and 509 nm were used, respectively. To monitor tryptophan fluorescence, excitation and emission wavelengths of 280 and 335 nm were used, respectively.

### Cell lines for flow cytometry and the luciferase signaling assay

HEK293 cells containing an NF-κB luciferase reporter were transfected with mGITR, the mGITR-swap mutant (K78R, Y80H, D93K, I94F, V95S, and R99Q), or hGITR in the pcDNA3.1 plasmid with a geneticin selectable marker. Transfected cells were maintained in Dulbecco’s modified Eagle’s medium supplemented with 10% heat-inactivated fetal bovine serum, 25 mM glucose, 6 mM l-glutamine, penicillin (100 U/ml), streptomycin (100 μg/ml), hygromycin (100 μg/ml), and G418 sulfate (600 μg/ml) and cultured at 37°C and 5% CO_2_.

### Flow cytometry

For experiments comparing binding of DTA-1 IgG to mGITR and mGITR-swap, HEK293 NF-κB luciferase reporter cells transfected with either full-length mGITR or mGITR-swap were incubated with DTA-1 (0.5 μg/ml) (Bio X Cell, catalog number BP0063), or mGITR was labeled with no primary antibody for the negative control. Following, the cells were treated with fluorescein isothiocyanate (FITC)–conjugated mouse anti-rat IgG2b secondary detection antibody (0.5 μg/ml) (BD Biosciences, catalog number 553884) for 30 min on ice. For experiments comparing binding of Fab and IgG to mGITR or hGITR, HEK293 NF-κB luciferase reporter cells transfected with either mGITR or hGITR were incubated with no primary antibody (negative control) or with DTA-1 IgG (human IgG1 subclass prepared as described), DTA-1 Fab, TRX518 IgG, or TRX518 Fab (1 μg/ml) for 30 min on ice. Excess IgG or Fab was washed out, and FITC-conjugated secondary goat anti-human Fab-specific antibody (1 μg/ml) (Sigma-Aldrich, catalog number F5512) was added and incubated for 30 min on ice. Dead cells were excluded using the Zombie NIR Fixable Viability Kit (BioLegend, catalog number 423105). Data were acquired on the Cytek Biosciences Aurora Spectral Flow Cytometer and analyzed using FlowJo software.

### Luciferase signaling assay

HEK293 NF-κB luciferase reporter cells transfected with either mGITR or hGITR were plated in 96-well clear-bottom black-wall plates at 10,000 cells per well and allowed to adhere overnight. The following day, mGITR-expressing cells were treated with DTA-1 Fab at 134 nM (6.67 μg/ml) or DTA-1 IgG at 67 nM (10 μg/ml), and hGITR-expressing cells were treated with TRX518 Fab at 134 nM (6.67 μg/ml) or TRX518 IgG at 67 nM (10 μg/ml). Untreated cells were used as a control. Cells were incubated with their treatments for 5 hours at 37°C, and luciferase signal was measured using the ONE-Glo Luciferase Assay Kit (Promega) per the manufacturer’s instructions. DTA-1 IgG and Fab were generated as described (see the “Expression and purification of DTA-1 and generation of DTA-1 Fabs” section). TRX518 IgG was provided by Leap Therapeutics, and Fab fragments were generated by papain cleavage and purified using the Pierce Fab Preparation Kit (Thermo Fisher Scientific).

### Statistics

Statistical differences were calculated using GraphPad Prism (GraphPad Software Inc.). One-way analysis of variance (ANOVA) was used, followed by a post hoc Tukey’s multiple comparison test for comparison between more than two groups. Alpha was set at 0.05, and *P* values less than 0.05 were considered statistically significant.

## Notes

View/request a protocol for this paper from *Bio-protocol*.
