# Supplementary material for: Therapeutic antibody activation of the glucocorticoid-induced TNF receptor by a clustering mechanism
Source: Sci Adv. 2022 Feb 25;8(8):eabm4552. doi: 10.1126/sciadv.abm4552 (PMC8880771; doi:10.1126/sciadv.abm4552)
Supplement: Supplementary file 1 — Figs. S1 to S4 Table S1 [file sciadv.abm4552_sm.pdf]

Science Advances
AAAS

Supplementary Materials for

Therapeutic antibody activation of the glucocorticoid-induced TNF receptor by a clustering mechanism

Changhao He, Rachana R. Maniyar, Yahel Avraham, Roberta Zappasodi, Radda Rusinova, Walter Newman, Heidi Heath, Jedd D. Wolchok, Rony Dahan, Taha Merghoub*, Joel R. Meyerson*

*Corresponding author. Email: merghout@mskcc.org (T.M.); jrm2008@med.cornell.edu (J.R.M.)

Published 25 February 2022, *Sci. Adv.* **8**, eabm4552 (2022)
DOI: 10.1126/sciadv.abm4552

This PDF file includes:

Figs. S1 to S4
Table S1

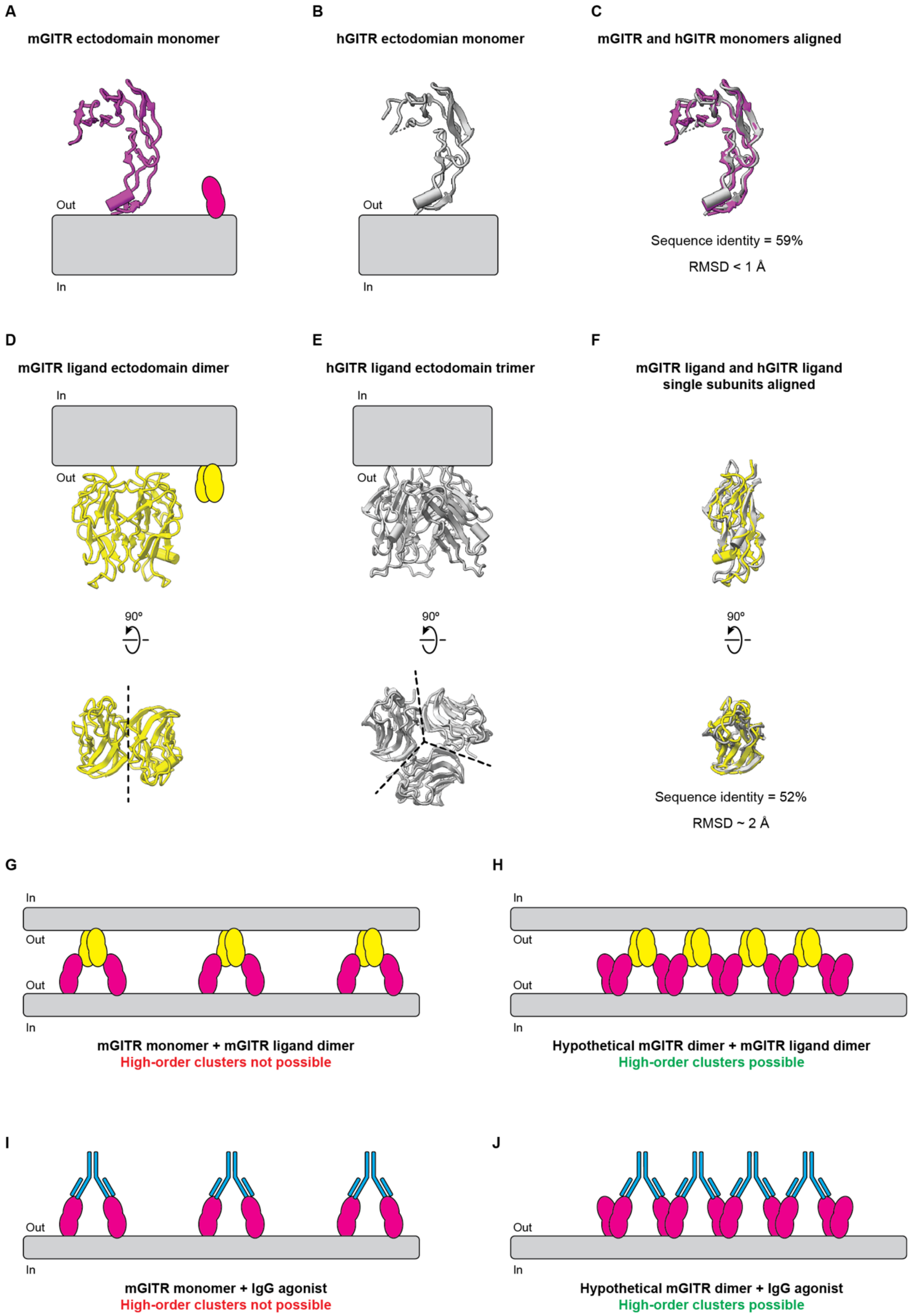
A
mGITR ectodomain monomer
Out
In
B
hGITR ectodomian monomer
Out
In
C
mGITR and hGITR monomers aligned
Sequence identity = 59%
RMSD < 1 Å
D
mGITR ligand ectodomain dimer
In
Out
90º
E
hGITR ligand ectodomain trimer
In
Out
90º
F
mGITR ligand and hGITR ligand single subunits aligned
90º
Sequence identity = 52%
RMSD ~ 2 Å
G
In
Out
Out
In
mGITR monomer + mGITR ligand dimer
High-order clusters not possible
H
In
Out
Out
In
Hypothetical mGITR dimer + mGITR ligand dimer
High-order clusters possible
I
Out
In
mGITR monomer + IgG agonist
High-order clusters not possible
J
Out
In
Hypothetical mGITR dimer + IgG agonist
High-order clusters possible

Fig. S1. Overview of structure and clustering for GITR and GITRL.

(A) Structure of the mouse GITR ectodomain monomer (PDB: 7KHX). Also depicted as illustration. **(B)** Structure of the human GITR ectodomain monomer (PDB: 7KHD). **(C)** Structural alignment of the mouse and human GITR ectodomain monomers. **(D)** Structure of the mouse GITR ligand ectodomain dimer (PDB: 7KHX). Also depicted as illustration. **(E)** Structure of the human GITR ligand ectodomain trimer (PDB: 7KHD). **(F)** Structural alignment of individual subunits from the mouse and human GITR ligand oligomers. **(G)** Illustration of mGITR monomers (magenta) interacting with mGITR ligand dimers (yellow). **(H)** Illustration of hypothetical mGITR dimers (magenta) interacting with mGITR ligand dimers (yellow). A receptor dimer would facilitate formation of high-order receptor arrays when interacting with the ligand dimer. **(I)** Illustration of mGITR monomers (magenta) interacting with DTA-1 IgG (cyan). **(J)** Illustration of hypothetical mGITR dimers (magenta) interacting with DTA-1 IgG (cyan). A receptor dimer would facilitate formation of high-order receptor arrays when interacting with IgG.

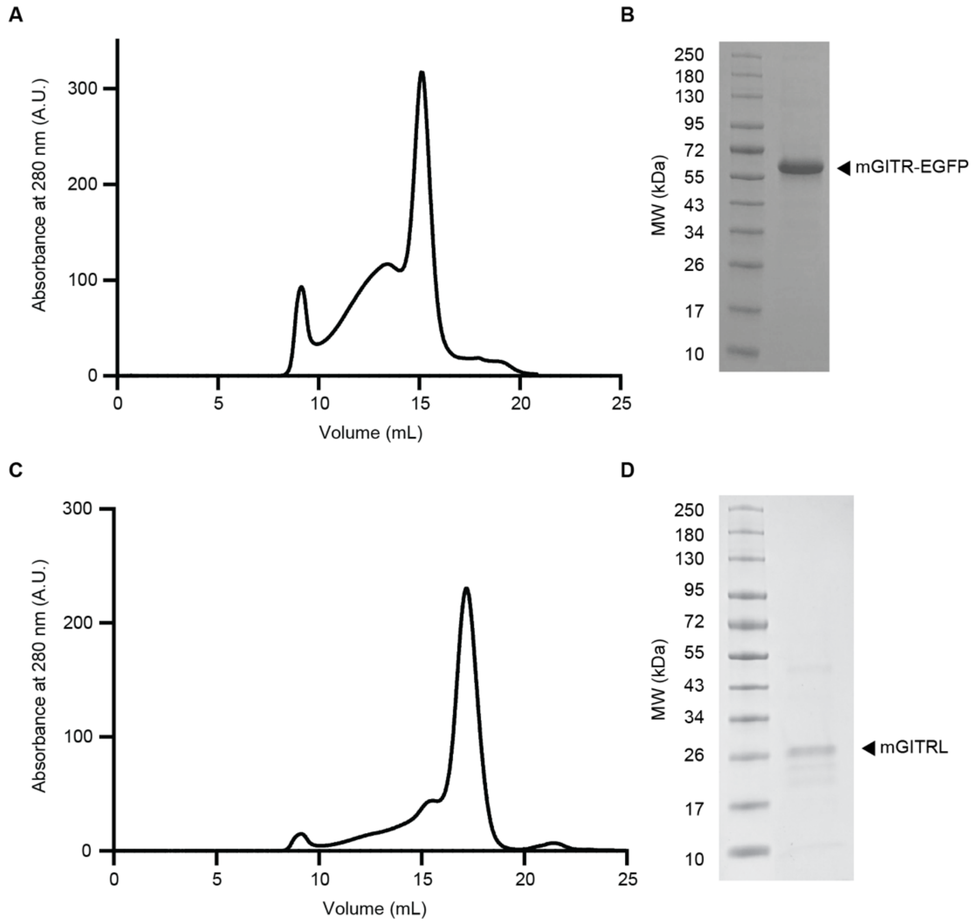

Fig. S2. Purification of full-length mGITR and mGITRL.

(A, B) Size-exclusion chromatography trace and SDS-PAGE of full-length mGITR fused to a C-terminal EGFP and an octa-Histidine affinity tag. **(C, D)** Size-exclusion chromatography trace and SDS-PAGE of full-length mGITRL fused to a Strep affinity tag. SDS-PAGE shows the peak fractions from gel size-exclusion chromatography.

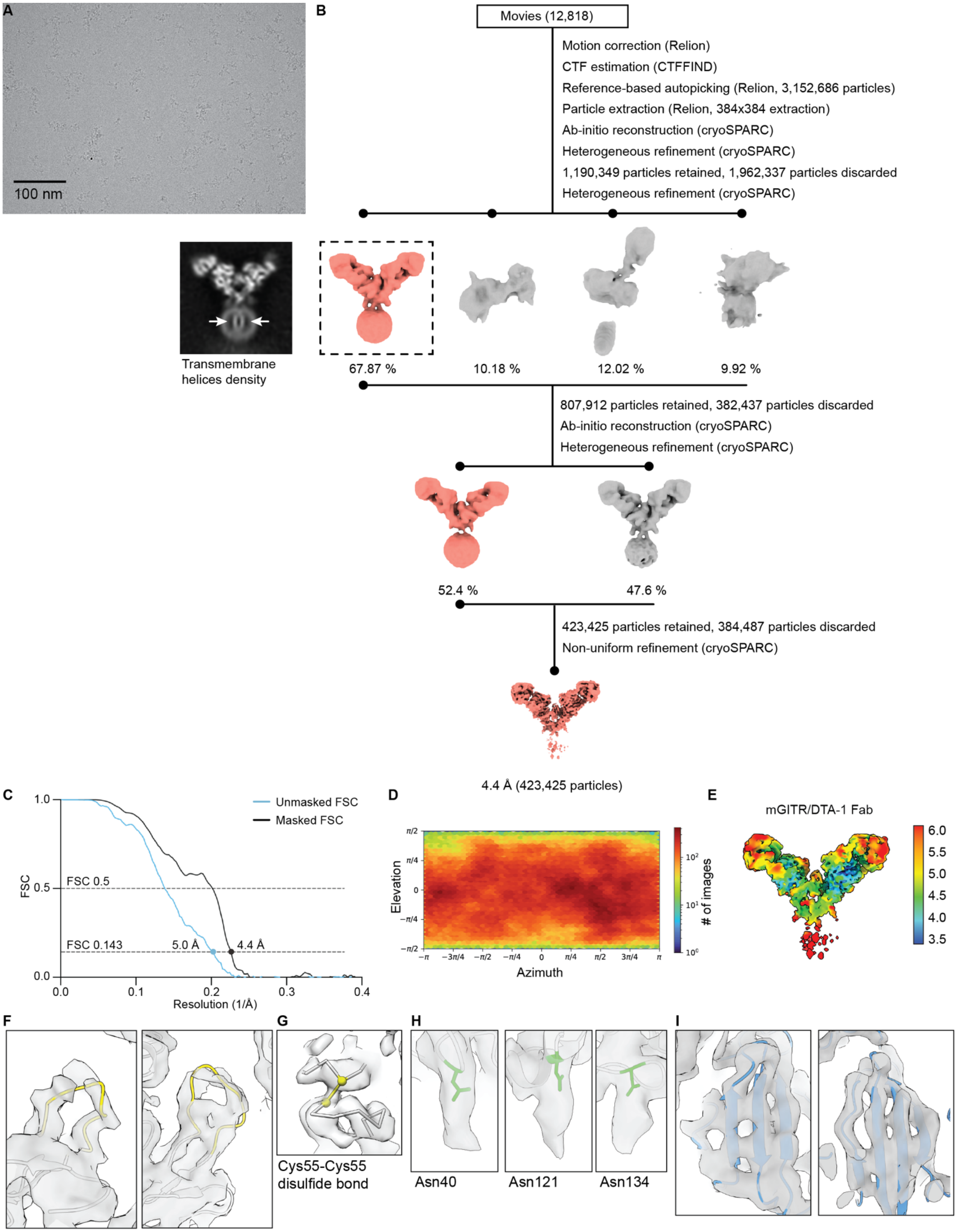
A
100 nm
B
Movies (12,818)
Motion correction (Relion)
CTF estimation (CTFFIND)
Reference-based autopicking (Relion, 3,152,686 particles)
Particle extraction (Relion, 384x384 extraction)
Ab-initio reconstruction (cryoSPARC)
Heterogeneous refinement (cryoSPARC)
1,190,349 particles retained, 1,962,337 particles discarded
Heterogeneous refinement (cryoSPARC)
Transmembrane helices density
67.87 %
10.18 %
12.02 %
9.92 %
807,912 particles retained, 382,437 particles discarded
Ab-initio reconstruction (cryoSPARC)
Heterogeneous refinement (cryoSPARC)
52.4 %
47.6 %
423,425 particles retained, 384,487 particles discarded
Non-uniform refinement (cryoSPARC)
4.4 Å (423,425 particles)
C
Unmasked FSC
Masked FSC
FSC 0.5
FSC 0.143
5.0 Å
4.4 Å
FSC
Resolution (1/Å)
D
Elevation
Azimuth
# of images
E
mGITR/DTA-1 Fab
6.0
5.5
5.0
4.5
4.0
3.5
F
G
Cys55-Cys55 disulfide bond
H
Asn40
Asn121
Asn134
I

Fig. S3. Cryo-EM image processing workflow for the mGITR/DTA-1 Fab structure.

(A) Cryo-EM micrograph of mGITR with DTA-1 Fab. **(B)** Data processing workflow for mGITR with DTA-1 Fab. The workflow proceeds from top to bottom. Cryo-EM density maps are color-coded as orange or gray according to whether they are retained or discarded, respectively. The percentage of particles in each class from heterogeneous refinement is given. The grayscale image is a slice through the three-dimensional class average which is presented with a dashed bounding box, and highlights the two mGITR transmembrane helices which are visible at low resolution during early stages of refinement. The helices are not visualized in later stages because the refinement is dominated by the extra-cellular region of mGITR. **(C)** Gold-standard Fourier shell correlation (FSC) curves for the unmasked map (cyan) and masked map (black). **(D)** Angular distribution plot for particles in the reconstruction, as generated by cryoSPARC. **(E)** Local resolution heat map with color bar units in Å. **(F)** Cryo-EM density for apical loops on mGITR subunit A (left) and B (right). The densities are fitted with the molecular model for mGITR, and the apical loop is colored yellow. **(G)** Cryo-EM density for apical loop interface shown with Cys55 as yellow spheres (both subunits) and disulfide bond in yellow. The perspective is from the extracellular space looking “down” at the “top” of the receptor subunit interface. **(H)** Cryo-EM densities for N-linked glycans observed in the mGITR structure (Asn40, Asn121, Asn134 from left to right). Each Asn residue is highlighted in green. **(I)** Representative beta sheet cryo-EM densities from the DTA-1 Fab, with the molecular model colored in blue.

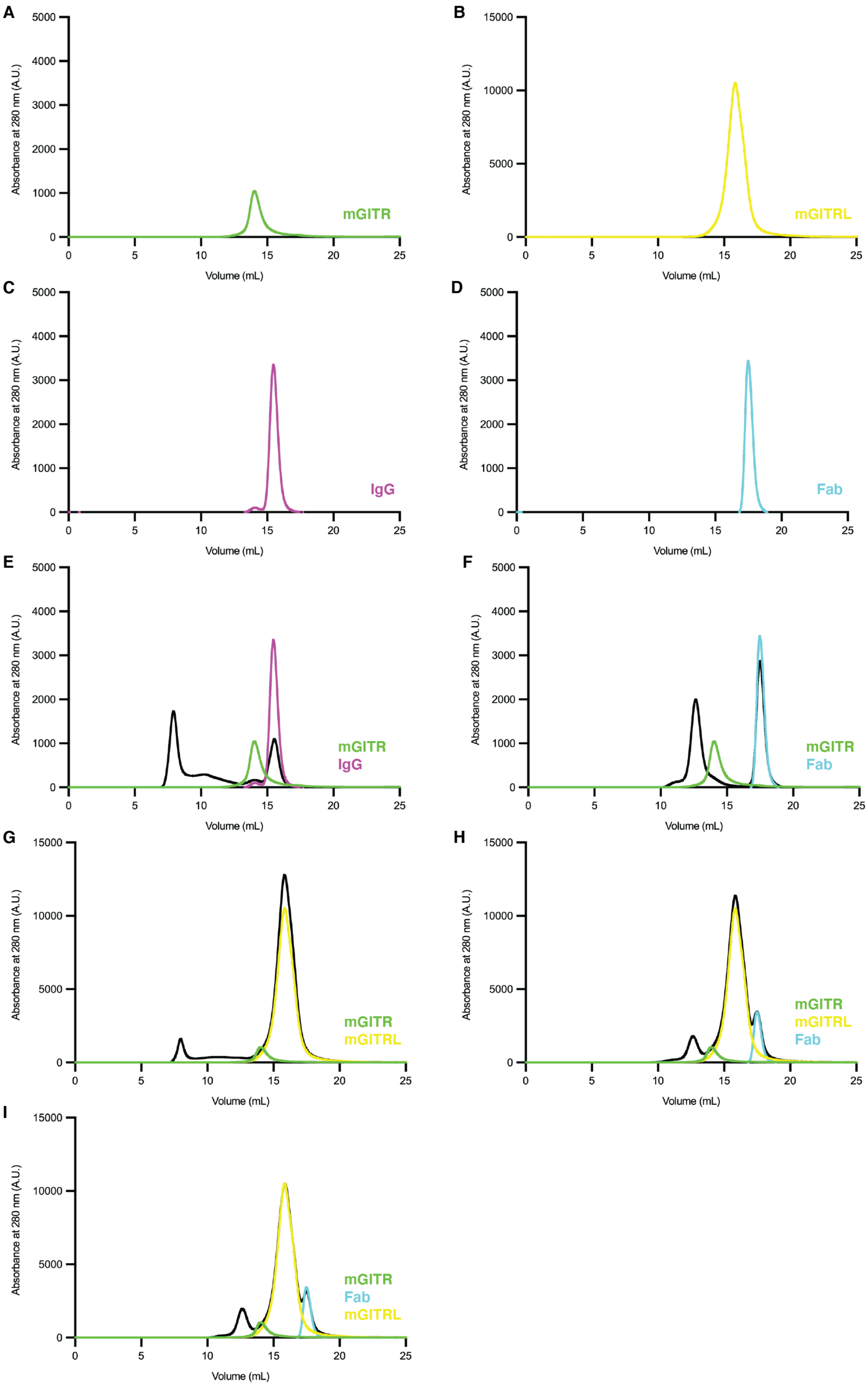
A
B
C
D
E
F
G
H
I
Absorbance at 280 nm (A.U.)
Volume (mL)
mGITR
mGITRL
IgG
Fab

Fig. S4. Chromatographic binding controls using Tryptophan fluorescence.

(A-I) Fluorescence size exclusion chromatography of $mGITR_{egfp}$, mGITRL, DTA-1 Fab, and DTA-1 IgG using excitation and emission wavelengths of 280 nm and 335 nm, respectively. This protocol exploits the inherent fluorescent properties of Tryptophan and is thus independent of the presence or absence of EGFP. The traces for $mGITR_{egfp}$ (green), mGITRL (yellow), DTA-1 IgG (magenta), and DTA-1 Fab (cyan) are colored consistently and as indicated in all panels. Each black trace shows the result of mixing the proteins in each panel. The traces show (A) $mGITR_{egfp}$ alone, (B) mGITRL alone, (C) DTA-1 IgG alone, and (D) DTA-1 Fab alone. Also shown are (E) $mGITR_{egfp}$ with DTA-1 IgG, (F) $mGITR_{egfp}$ with DTA-1 Fab, (G) $mGITR_{egfp}$ with mGITRL, (H) $mGITR_{egfp}$ incubated (5 min, 4°C) with mGITRL before adding DTA-1 Fab, and (I) $mGITR_{egfp}$ incubated with DTA-1 Fab before adding mGITRL.

	mGITR with DTA-1 Fab<br>EMDB-24444<br>PDB-7RFP
**Data collection and processing**	
Magnification	36,000
Voltage (kV)	200
Electron exposure (e–/Å^2)	53.10 - 57.04
Defocus range (μm)	1.1 - 2.9
Pixel size (Å)	0.548
Pixel size for processing (Å)	1.096
Box size (pixels)	384
Symmetry imposed	C1
Initial particle images (#)	3,152,686
Final particle images (#)	423,425
Map resolution (Å)	4.4
FSC threshold	0.143
Map resolution range (Å)	3.5 - 6.0
**Refinement**	
Clashscore, all atoms	3.23
Protein Geometry	
Poor rotamers	8.00%
Favored rotamers	88.00%
Ramachandran outliers	2.99%
Ramachandran favored	87.48%
MolProbity score	2.41
Cβ deviations >0.25Å	0.78%
Bad bonds	0.07%
Bad angles	0.27%
Peptide Omegas	
Cis Prolines	9.09%
Cis nonProlines	0.00%
Twisted Peptides	0.00%
Low-resolution Criteria	
CaBLAM outliers	5.40%
CA Geometry outliers	2.48%

Table. S1. Cryo-EM data collection and refinement table.
